# An assessment of priority setting process and its implication on availability of emergency obstetric care services in Malindi District, Kenya

**DOI:** 10.11604/pamj.2015.22.156.7296

**Published:** 2015-10-20

**Authors:** Lilian Nyamusi Nyandieka, Yeri Kombe, Zipporah Ng'ang'a, Jens Byskov, Mercy Karimi Njeru

**Affiliations:** 1 Centre for Public Health Research, Kenya Medical Research Institute (KEMRI) Nairobi, Kenya; 2 Institute of Tropical Medicine and Infectious Diseases, Department of Public Health - Jomo Kenyatta University of Agriculture and Technology, Nairobi, Kenya; 3 Research Unit for Human Parasitology and the Environment, Faculty of Health and Medical Sciences, University of Copenhagen, Dyrlægevej 100, DK-1870 Frederiksberg C, Denmark

**Keywords:** Priority setting, decision making, EmOC, stakeholders, accountability

## Abstract

**Introduction:**

In spite of the critical role of Emergency Obstetric Care in treating complications arising from pregnancy and childbirth, very few facilities are equipped in Kenya to offer this service. In Malindi, availability of EmOC services does not meet the UN recommended levels of at least one comprehensive and four basic EmOC facilities per 500,000 populations. This study was conducted to assess priority setting process and its implication on availability, access and use of EmOC services at the district level.

**Methods:**

A qualitative study was conducted both at health facility and community levels. Triangulation of data sources and methods was employed, where document reviews, in-depth interviews and focus group discussions were conducted with health personnel, facility committee members, stakeholders who offer and/ or support maternal health services and programmes; and the community members as end users. Data was thematically analysed.

**Results:**

Limitations in the extent to which priorities in regard to maternal health services can be set at the district level were observed. The priority setting process was greatly restricted by guidelines and limited resources from the national level. Relevant stakeholders including community members are not involved in the priority setting process, thereby denying them the opportunity to contribute in the process.

**Conclusion:**

The findings illuminate that consideration of all local plans in national planning and budgeting as well as the involvement of all relevant stakeholders in the priority setting exercise is essential in order to achieve a consensus on the provision of emergency obstetric care services among other health service priorities.

## Introduction

Maternal mortality remains a major challenge to health systems around the globe [1] despite global efforts to improve the state of maternal health worldwide. Initiatives have been launched dating as far back as 1987 with the Safe Motherhood Initiative [2] and the Millennium development goals in 2000 [3]. In 2013, 289 000 women died during and following pregnancy and childbirth. Almost all of these deaths occurred in low-resource settings, and most could have been prevented as the health-care solutions to prevent or manage complications are well known [4]. The pregnancy-related complications can be prevented through improved access to adequate health-care services and Emergency Obstetric Care (EmOC). EmOC is a standard set of medical interventions which treat obstetric complications and, accordingly, prevent maternal deaths [5]. The importance of EmOC has been endorsed by most international health organisations, including WHO, UNICEF and UNFPA [6]. In Kenya, access to health care varies widely throughout the country and is determined on numerous factors, though in particular, major divides exist between rural and urban communities [7]. The availability of EmOC being better in cities and towns than in the rural areas can be explained by a number of factors including prioritization by governments of resources for hospitals over lower level facilities, difficulty of maintaining equipment and supplies in relatively more rural locations, and difficulty in retaining qualified staff in smaller facilities. In addition, government regulations and policies often make it difficult for a facility without a physician present to perform certain functions [8]. It is estimated that for every woman who dies in childbirth, another 20-30 women suffer serious injury or disability due to complications during pregnancy or delivery [9]. The problem is partly driven by lack of access to quality maternal health services, and though health sector infrastructure has grown over the past decade, [10] many women still live at a considerable distance from health facilities [11]. A study conducted in Malindi to assess the actual existence and functionality of EmOC services found that there were geographical inequities in distribution of EmOC facilities in the district [12] and that some of the facilities classified as basic EmOC did not qualify as they did not meet all the requirements to be classified as such [5]. This has enormous implications for access to care for women living in rural areas. In the circumstances, this calls for priority setting to address maternal health issues and especially EmOC service delivery in the rural areas.

Priority setting can be defined as the distribution of resources among competing programs or people [13]. In essence, as there are more claims on resources than the actual resources available, some form of priority setting must occur [14]. Arguably, this is most important when resources are scarce, as is the case in low-income countries [15]. This rationing is a complex and difficult problem faced by all decision makers at all levels of all health systems [16]. Globally, the debate on priority setting in health service delivery to a large extent involves the government as an allocator of scarce health care resources. This entails the selection of health services, programmes or actions that will be provided first, with the purpose of improving the health benefits and distribution of health resources [17, 18]. Hence, policy makers need to make important decisions on the use of public funds, which disease areas to target, populations and with which interventions. Often, these choices are not based on rational and transparent process, and resources may not be used to an optimal extent [19, 20]. Various models of decision making have been proposed. However, the Accountability for Reasonableness (AFR) stands out as an ethical framework for fair priority setting [21] which was developed in the context of real world priority setting process [22] and has been used to study actual priority setting processes [23]. According to AFR, a fair priority setting process meets four conditions namely relevance, publicity, appeals/revision and enforcement. The relevance criteria requires that the rationales for priority setting rest on reasons (evidence and principles) that fair minded people can agree are relevant in the context. Closely linked to this condition is the inclusion of a broad range of stakeholders in the decision making process [24]. This ensures that a wide range of values and principles are taken into account. The publicity condition requires that priority setting decisions and their rationales be publicly accessible as justice cannot abide secrets where people's wellbeing is concerned. For the appeals/revision condition, there must be a mechanism for challenge, including the opportunity for revising decisions in light of considerations that stakeholders may raise. In enforcement, there is either voluntary or public regulation of the process to ensure that the first three conditions are met [21]. This study assessed priority setting process and its implication on availability, access and use of EmOC services in Malindi. This paper describes the priority setting process at the district and its implication on availability of EmOC services.

## Methods

### Study design

Qualitative approach was considered most appropriate to answer the research questions on experiences and priority setting processes in EmOC service provision. To strengthen the credibility of the study findings, triangulation of data sources and methods was used. This included documentary reviews, in-depth interviews (IDIs) and focus group discussions (FGDs) to collect information.

### Study area and setting

This study was conducted in Malindi district (now a sub-county of Kilifi County) in the coastal region of Kenya between November 2012 and April 2013. This was a follow up of an EU-funded five year intervention study “REsponse to ACcountable priority setting for Trust in health systems” (REACT). EmOC was one of the domains within the REACT project that was evaluated. During the time the study was conducted, the study site comprised of Malindi and Magarini, which have since been split into Malindi and Magarini Sub-counties. The area had 105 public and private health facilities [25]. There were three Comprehensive EmOC facilities, one public, and two private facilities, all located in Malindi town [12]. This study was conducted in six facilities from different sites, purposively selected with the assistance of the District Medical Officer of Health (DMOH). Among these, four were public health facilities; one, a health centre in level three and three dispensaries in level two. In addition, one faith based dispensary and two NGO run facilities were selected for the study. These facilities were selected due to the distance between them and the main referral facility in Malindi which pose a challenge in accessing maternal health services whenever there is a childbirth complication. The selected facilities are between 37km and 60km from the referral facility (12).

### Study population

The informants consisted of facility in-charges, reproductive health services heads, stakeholders and partners who provide and support maternal health services and health facility committee members who represent the community. At the community level, women, married men, traditional birth attendants (TBAs), opinion leaders and Community Health Workers (CHWs) were included in the study.

### Recruitment

Facility in-charges and heads of reproductive health services were included in the study by virtue of their positions at the facilities as planners and providers of maternal health services. Stakeholders, partners, faith based and non-governmental institutions were selected to share their views and experiences on maternal health issues with the community; and their support for priorities set for the public health system. Committee members were selected as community representatives. Women seeking health services were invited to give an interview and those who agreed were included in the study as end users; TBA group leaders and CHWs in the area were identified and included in the study to share their experiences with the community members. Opinion leaders were selected with the help of the facility in-charges to share their views on the topic. Male and female informants were recruited for FGDs with the help of opinion leaders in the areas. Men were included in the study as family decision makers.

### Ethical approval

Approval to conduct this study was granted by the Ethical Review Committee of Kenya Medical Research Institute (Scientific Steering Committee No. 2288). Permission to conduct the study at the health facilities was granted by the then DMOH while written consent was given by the informants both at the facility and community levels. Permission to audio record the interviews was sought from each informant.

### Data collection

A total of 22 IDIs and seven FGDs were conducted. IDIs were conducted with facility in-charges, reproductive health services heads, stakeholders and health facility committee members. Women seeking services at the facilities, TBAs, CHWs and opinion leaders were also interviewed. Three FGDs were conducted with male members as decision makers; three with women as the end users of delivery services and one with TBAs. Interview guides addressed the responsibilities of the interviewees, their understanding of priority setting, the process, maternal health issues in the district and their local settings, involvement of stakeholders, implementation of decisions in the AOPs, utilisation of the existing facilities, distribution of EmOC facilities and on what they do when they have women with delivery complications. The AFR conditions were factored in the guides. Specific questions regarding priorities in maternal health programs were directed to stakeholders and partners. These interviews included the kind of services offered and whether any special arrangements were in place with the existing public health system. Interviews with community members were on their experiences while seeking health services at the facilities, their role in priority setting and their perceptions towards priority setting for EmOC services. FGDs centered on challenges of access and utilisation of services by the service consumers; and decision making at the family level to seek EmOC services. The health personnel, stakeholders and partners were interviewed at their respective places of work. Committee members were interviewed at the health facilities where they are members while women were interviewed at the health facilities where they were seeking services. TBAs, CHWs, and opinion leaders were interviewed in their local health facility while FGDs were held at places that were convenient for the informants. Two health personnel and two committee members were not able to create time to participate; and two women declined because they did not have permission from their husbands to participate. Data collection was concluded when the target population had been covered. In total 15 documents were reviewed which included district health plans, national policy documents, guidelines and local publications. This review was carried out to facilitate further understanding of the priority setting process at the district.

### Data analysis

Data was analysed thematically. The process of identification of concepts was introduced as the data collection exercise continued. Guides were revised as new information was introduced in the study. Once all the data collection was finalized, it was transcribed verbatim. Interviews conducted in Kiswahili were translated into English. Transcribed data was given code numbers for anonymity. Data was explored to identify important and relevant themes of the study. These were subsequently labeled according to their relevance and a series of categories built up to explain the events that were emerging from the study. Emerging categories were merged to form core categories which are discussed in this paper. Categorization was done manually.

## Results

This section describes core categories relating to priority setting for EmOC services. The themes reported here are in regard to what the informants understood by priority setting, the process at facility and district levels, stakeholders’ involvement and communication of information to the community.

### Meaning of priority setting

Majority of the health personnel understand priority setting to mean handling or addressing the most pressing issues first while others can wait. *“Priority setting I think is how we organize how we give service, you know we have so many needs, but there are those that are urgent and there are those that can at least wait. Therefore, the urgent need is given first priority” - Reproductive health services head, facility 2*.

*“Priority setting is taking the most urgent things to be handled first.… that this is more urgent than the other or this is more important, this is what we want to achieve at a given time” - Reproductive health services head, facility 6*.

### Priority setting process

#### Facility level

The priority setting process starts at the facility level. The heads of facilities review data from the previous year and use the information to set targets for the following year. They incorporate the challenges they face in the current year in order to sort them out in the following financial year. However, it emerged that there was no uniformity in the way the heads of the facilities prepared their work plans.

*“What we normally do, we set the targets according to the performance contract, maybe like last year, we had a certain number of clients; we normally get the targets based on the population. And maybe we want to increase it by a certain percentage as a facility. So we don't normally sit in the committee and discuss” - In-charge, facility 1*.

*“We have some forms here that we have to record at the end of the month for the activities to be carried out by the facility; and some minutes from the meetings. And we have targets which we prepare and share with the district and show how well we shall achieve these targets” - Reproductive health head, facility 4*.

#### District level

Priority setting process at the district level is guided by the national objectives. The District Health Management Team (DHMT) members sign a performance contract with the Permanent Secretary, being the ones in charge of the running of the district health affairs. DHMT members supervise facilities to ascertain that they work according to their work plans because the actual implementation of what is signed for happens at the facility level. At the end of every year, facility heads from both public and private facilities are invited to the district headquarters for a target setting meeting where they share and deliberate on data and work plans from their facilities. These work plans are then used to draft Annual Operation Plans (AOPs) for the district. A review of the Ministry of Health (MoH) guidelines for management boards and committees collaborates this information.

*“There is a performance contract which we sign, so from the national level we are guided by whatever objectives we are supposed to meet. So they narrow down to the district level, and to the facility level, so you can't take a priority outside what the PS (Permanent Secretary) is supposed to achieve” - Reproductive health services head, facility 6*.

### Stakeholders involvement in the decision making process

Only the health personnel participate in the decision making process; and they feel that the relevant stakeholders such as community members are not sufficiently involved in the process. The informants say the stakeholders are many. Consequently, bringing them all on board is a challenge the facilities cannot handle due to time and other constraints beyond them. However, MoH guidelines highlight the involvement of more stakeholders including community members through committee representatives.

*“Community members are not sufficiently represented in decision making about issues that affect them….” - Reproductive health services head, facility 4*.

*“The agenda of meetings follow the progressive report; other agendas come from the facility head in case they have something new to report” - Committee representative facility 5*.

*“They have never involved us in their sittings to know what our problems are”- Female informant, FGD facility 5*.

### Communication of information to the community

The findings indicate that different modes of communication are used to pass information and decisions arrived at to the community. Some facilities use Public Health Officers, committee members, CHWs, while others send information through the TBAs. The information can be relayed to the community directly or communicated through the chiefs’ barazas (meetings organized by chiefs who are in charge of a location, which is an administrative unit) whenever they are held. Special chiefs’ barazas can be organized if the information to be passed is urgent.

*“Elders mobilize people to attend barazas, but then you can get nine men and 70 women in a meeting. Men send their wives to represent them, and the women may not relay the message from the meetings. Or they simply don't understand what the meeting was all about”- Committee representative, facility 5*.

*“If we have very urgent information or decision it has to pass through the committee to the community” - in-charge, facility 5*.

*“It's easier for the message to reach the community through the CHWs than the DHCs (Dispensary Health Committee) because the CHWs represents few households, like 15. They can do it better within a short time, than the DHC who have the whole village” - In-charge, facility 1*.

## Discussion

This paper documents findings on the priority setting process and its implication on availability of EmOC services in Malindi. The findings are also discussed in consideration with the Accountability for Reasonableness (AFR) conditions. Countries with very different health care systems and levels of health care are all grappling with the problem of how to reconcile growing demands and constrained resources [19]. There is limited literature reporting actual priority setting in low-income countries [26] which indicates that priority setting occurs by chance [27] and that there is need to assist decision makers with priority setting [28, 29] to improve and make proper use of available data to inform decisions [30]. Two key findings emerge clearly from this study. One, that priority setting is not an all-inclusive matter and two; the priority setting process does not include the infrastructural development and distribution of facilities in the district. This study established that there is constrained freedom in setting priorities; with the process largely dependent on guidelines from the national level. Only the health personnel are involved in the decision making process, negating the AFR condition of relevance which requires the inclusion of a broad range of stakeholders. Any other stakeholders’ involvement goes only as far as supporting decisions that have been arrived at by the health personnel. The end users are not directly involved in decision making; though this is implied when community members are included in the facility health committees [31]. Facility committee members do not participate in the planning meetings at the district headquarters, where the decision making exercise takes place. However, they sit in meetings to approve and oversee facility expenditure. Meanwhile, involvement of communities and other relevant stakeholders feature prominently in policy documents and guidelines, but the existing mechanisms and processes for decision making at district level has substantial shortcomings in terms of participation in actual practice [32–34]. From the findings, it appears that there is an attempt to observe the publicity condition, though the method through which it is practiced has shortcomings. Decisions arrived at are shared with the community members mainly through *barazas*, where the bearers of the information attend and address those present. The challenge with the *barazas* as observed by the informants is that the women who attend may not relay this information to their husbands. There is also the possibility that information delivered by individuals from house to house may not reach all it was intended for. Scholars have argued the need for public response to enhance the legitimacy of priority setting decision making [24, 35, 36]. Members of the public can adopt various roles to participate in priority setting such as tax payers, collective decision makers or as patients [24]. However, members of the public have sometimes been reluctant to support public participation in health care priority setting as they feel unqualified to participate [24, 37–39]. In the case of Malindi, it is evident that the public is not given an opportunity to participate and therefore cannot appeal for revision of decisions that are arrived at as there is no platform to do so. The enforcement condition requires that there is either voluntary or public regulation of the process to ensure that the first three conditions are met. In this case, it is not possible to enforce that which does not exist. The priority setting process is routine and closed to an extent that even though the participants feel that there is need to involve other stakeholders, they cannot do anything as the decision to involve the others does not rest with them.

### Resource allocation for priority implementation

Under-financing of the health sector remains a pervasive challenge for health service delivery, considering that it reduces the health sector's ability to ensure an adequate level of service provision to the population [40]. In Kenya, funds allocated to the facilities are not based on budgeted plans for service delivery but on available resources from the Ministry of Finance (MoF) [41]. Documentary reviews indicate that districts receive as little as half the funding they budget for. And allocations have strict guidelines tied to the expenditure. This could be a possible explanation as to why there are priorities that feature in the AOPs which have not been implemented for up to three years consecutively [42–44]. There is thus, little relationship between plans, available funds and actual implementation [45]. Further, current budgeting practices have delays in allocation and disbursement of funds to the district level, thereby weakening control over finances at the district level [46] and consequently impeding implementation of decisions arrived at.

### Implication on availability of EmOC service

Though the physical infrastructure for health in Kenya has expanded since independence, the most crucial factor influencing both the quantity and quality of health care services to be delivered is the planning of where health facilities will be constructed and the types of services they will offer. Capital investments in new public facilities or in the rehabilitation of old ones have substantial long term repercussions upon the recurrent budget of the MoH [45]. A review of the district health plans reveals that plans to upgrade existing health facilities are through the contribution of the community which takes long to accomplish [42, 44]. In the study area, new facilities were reported to have been put up by the local area politicians. These facilities are not operational as they do not have resources to function, bringing into play a disconnect between the legislators and decision makers. It is probable that the non-involvement of relevant stakeholders including community members in planning and decision making could be responsible for the gaps in availability and poor distribution of the EmOC facilities in Malindi. As Oakley [47] argues, community participation is a principal factor in the success of development programs, as it allows individuals to choose what they like or don't like. And even though professionals have downgraded those they consider lay persons in priority setting process [48], it is essential to find a forum in which community members can engage with other stakeholders in order to communicate their needs and concerns.

### Study Strengths

This study was conducted at the first point of contact for the service consumers and health providers; therefore, giving a clear reflection of what happens. This, unlike most studies which are conducted only at the district headquarters, presented a good opportunity to have a feel of the need of the consumers and health providers, making a strong point. It is likely that the current findings apply more widely than Malindi Sub-county only and that they are vital in providing the newly created county governments with findings that might directly affect them.

### Study limitations

Priority setting at the district level is only a part of a process. Many more players are involved at various higher levels at the MOH and MOF. The current study may not have captured all the priority setting processes and challenges at the district level mainly because the study was conducted at the lower levels of the referral health system. However, it is likely that these findings apply more widely to the other levels of the health system within the district.

## Conclusion

Priority setting process at the district level lacks the participation of important stakeholders such as community members. Resource allocation is not pegged on budgeted plans; and expenditure has strict guidelines which has the potential to hinder service provision. Therefore, consideration of all local plans in national planning and budgeting as well as the involvement of all relevant stakeholders in the priority setting exercise might considerably change the current status on availability of EmOC services in Malindi; and indeed other rural settings within the country.
